# PATIENT EXPERIENCES OF PHYSICAL ACTIVITY AND INACTIVITY IN THE STROKE UNIT: AN INTERVIEW STUDY

**DOI:** 10.2340/jrm.v56.19502

**Published:** 2024-02-08

**Authors:** Malin REINHOLDSSON, Gisela HERRANEN, Katharina S. SUNNERHAGEN, Annie PALSTAM

**Affiliations:** 1 Department of Clinical Neuroscience, Institute of Neuroscience and Physiology, Sahlgrenska Academy at the University of Gothenburg; 2 Department of Occupational Therapy and Physiotherapy, Sahlgrenska University Hospital; 3 Neurocare, Sahlgrenska University Hospital, Gothenburg and; 4 School of Health and Welfare, Dalarna University, Falun, Sweden

**Keywords:** exercise, interview, physical activity, sedentary behaviour, stroke, thematic analysis, qualitative research

## Abstract

**Objective:**

Stroke unit care is highly recommended after stroke, but patients in these units are often physically inactive. The aim of this study was to explore patient experiences of physical activity and inactivity in the stroke unit.

**Design:**

Qualitative interview study.

**Subjects:**

Sixteen participants with stroke; a heterogeneous sample with differences in sex, age, and stroke severity from 8 Swedish stroke units.

**Methods:**

In-depth interviews 1–2 weeks after discharge analysed using thematic analysis.

**Results:**

The analysis resulted in three themes: 1: Dealing with the challenges of a changed body while striving to become independent; 2: The stroke unit is crucial for physical activity; and 3: Physical activity is important for interaction with others, autonomy, and feeling seen. Participants described how they coped with a new situation when finding new ways to move and function. In addition, they wanted to be involved in their own stroke rehabilitation.

**Conclusion:**

The participants expressed the following experiences of being in the stroke unit: movement is more important than physical activity and involves being seen and respected; physical activity and exercise are necessary to achieve independence; process involvement is of importance to regain abilities; physical activity offers the possibility of choosing between community and being alone and influences the ability to connect with others and the outside world.

LAY ABSTRACT

Stroke unit care is considered best practice after stroke. However, patients tend to become inactive for reasons unknown. To describe physical activity and inactivity at the stroke unit from the patient perspective, 16 patients with stroke were interviewed. The interviews were analysed using a qualitative method, resulting in 3 themes: 1: Dealing with the challenges of a changed body while striving to become independent; 2: The stroke unit is crucial for physical activity; and 3: Physical activity is important for interaction with others, autonomy, and feeling seen. Participants expressed that movement means more to them than physical activity and involves being seen and respected. They described how they coped with a new situation when finding new ways to move and function. Participants expressed that physical activity is necessary to achieve independence and that they wanted involvement in their stroke rehabilitation. These results contribute with important insights into patient experiences that can be used to improve the quality of stroke unit care and rehabilitation.

Admission to stroke unit care is considered best practice for a patient after acute stroke (1) because a comprehensive stroke unit provides both acute care and early rehabilitation (2). Staff expertise, early mobilization, prevention of complications, and better diagnostic, care, and rehabilitation procedures are all aspects of comprehensive stroke unit care that contribute to better outcomes for patients after stroke (1).

In Sweden, 23,000 incident strokes occur every year, and 92% of patients are cared for in comprehensive stroke units (3). Rehabilitation begins at hospital admission, provided by multidisciplinary teams according to Swedish national guidelines. A person-centred approach is recommended in stroke rehabilitation, involving the patient as an active participant and establishing a partnership between the healthcare team and the patient (4). Although early rehabilitation is a key component in stroke unit care, many studies describe patients in these units as being physically inactive during their stays (5, 6). Physical inactivity is common after stroke and can increase the risk of recurrent cardiovascular incidents and reduced functional ability (7, 8). Physical activity is defined as body movement made by muscles that involves energy expenditure, and can be categorized into work-related, sports, training, household, or other activities of daily living (ADL) (9).

Patients in stroke units often spend most of their days in their patient rooms (73–89%), in bed (49–98%), or alone (56–60%), with variations among countries in Eastern and Western Europe and in Australia (10–13). Furthermore, patients in the stroke unit engage in physical activity 10–23% of the day, with an increase of 22 minutes spent sitting and 4 minutes spent upright per day at hospital (6, 13, 14). Factors related to physical inactivity in the stroke unit include stroke severity and advanced age (14). The built environment in hospitals can influence physical activity, with functional design of corridors, single or multi-bedrooms, placement of staff stations, and availability of communal areas and greens spaces (15). Physical activity in the stroke unit is promoted by meal service in communal areas and more time spent with physiotherapists (16). These known factors, however, can explain only in part why patients often are physically inactive when in the stroke unit (17). Although physical activity may reduce the risk of stroke recurrence and disability, physical activity levels often decrease after stroke. To date, no interview-based investigation has explored physical activity and inactivity for patients in stroke units. The aim of this study was to use a qualitative approach, based on analysis of patient interviews, to explore patient experiences of physical activity and inactivity in the stroke unit.

## METHODS

### Design

This qualitative study used in-depth interviews evaluated using a reflexive thematic analysis with an inductive approach (18). The underlying theory is social constructivism based on the assumption of relativism, which acknowledges multiple realities that vary depending on personal experiences and contexts (19). The design followed the COnsolidated criteria for REporting Qualitative research (COREQ) guidelines (20). The study was approved by the Swedish Ethical Review Authority, reference number 2021-06716-01. Oral and written informed consent was obtained from the participants.

### Patient participation

A patient research partner (GH) was engaged from the start of the study with collaboration on ethical application, project plan, and development of the interview guide. Furthermore, GH was engaged during the analysis process in discussing preliminary themes and revisions of themes to validate the interpretations of the participants’ descriptions in the analysis process. All participants received written and oral information about the study results. The Guidance for Reporting Involvement of Patients and the Public 2 (GRIPP2) reporting checklist was followed (21).

### Authors

MR is a 57-year-old woman, a PhD student in medicine with education in qualitative research methods, and a registered physiotherapist specialized in neurology with more than 30 years of clinical experience in stroke unit care. GH is a 57-year-old woman, an assistant nurse, and a patient research partner in this study with personal experience of having a stroke. KSS is a 66-year-old woman, a physician specialized in stroke, and a professor in rehabilitation medicine with almost 40 years of clinical and research experience in neurological diagnoses and extensive research experience, including in qualitative studies. AP is a 42-year-old woman and a registered physiotherapist with a PhD in medicine and vast experience in qualitative research. None of the authors had previous associations with the participants. The researchers contributed to knowledge production in this study and had awareness of their own preconceptions and experiences.

### Participants

Sixteen participants with stroke were recruited and participated in a single individual interview 1–2 weeks after discharge from the stroke unit. MR contacted the heads of departments at the 9 stroke units in Region Västra Götaland about participating in the recruitment of participants, and 8 units agreed. Inclusion criteria were patients with stroke, age 18 years or older, who stayed at a stroke unit ≥ 3 days and were Swedish speaking. Exclusion criteria were mobilization restrictions in the stroke unit or aphasia, cognitive impairment, or dementia that would hinder the interviews. The participants were purposively sampled with regards to variations in age, sex, and stroke severity, and their characteristics are described in Table I.

**Table I:** Characteristics of the study population (*n*=16)

	*n*	Median [IQR]	Min–max
Age, years		75 [10]	49–84
Men	7		
Women	9		
Pre-stroke frailty, Clinical Frailty Scale (1–9)		2.5 [3]	1–6
Pre-stroke physical activity (SGPALS) (1–4)		2 [1]	1–3
Inactivity	5		
Light physical activity	9		
Moderate physical activity	2		
Ischaemic stroke	14		
Haemorrhagic stroke	2		
Stroke severity, NIHSS on admission (0–42)		4 [7]	0–14
Length of stay (days)		10 [7]	5–39
Number of comorbidities		1.5 [3]	0–5
Discharged to their home	13		
Discharge to rehabilitation/short-term care	3		
Walked independently at discharge:			
Without walking aids	4		
With walking aids	9		
Wheelchair users	3		
Berg Balance Scale (0–56)	16	38 [22]	3–56
Barthel Index (0–100)	16	80 [19]	35–100
Timed Up and Go (s)	13	24 [13]	9–43
10-m optional speed (m/s)	13	1.6 [1.1]	9–26
10-m maximal speed (m/s)	12	1.1 [0.7]	7–16
6-minute walk test (m)	12	282 [184]	165–463

IQR: interquartile range; NIHSS: National Institute of Health Stroke Scale; SGPALS: Saltin Grimby Physical Activity Level Scale.

Involved physiotherapists working with patients in the stroke units recruited and tested the participants. Beforehand, the local physiotherapists had digital or face-to-face meetings with MR and received oral and written instructions about the study and recruitment process. The local physiotherapists administered oral and written information about the study and consent form to participants and assessed function during the first week after stroke. To ensure a study sample with a variation in characteristics MR was in contact with local physiotherapists on repeated occasions during the recruitment period. Data collected on participant characteristics were sex, age, stroke type, severity of stroke with the National Institutes of Health Stroke Scale (22), and comorbidities. Clinical assessments were conducted for descriptive purposes, including the Clinical Frailty Scale (23) for pre-stroke frailty, the Saltin-Grimby Physical Activity Level Scale for pre-stroke physical activity (24), the Barthel Index for personal care in everyday activities (25), Berg Balance Scale (26), and Timed Up and Go (27) for balance, as well as the 10-Metre Walk Test (self-selected and maximum speed) (28) and 6-Min Walk Test (29). There were no dropouts.

### Context

The interviews were conducted during September and October 2022 in the Swedish aftermath of the COVID-19 pandemic. At the time, patients were routinely tested for COVID-19 in the stroke units and were isolated if testing positive. Routines with face masks, visitation restrictions and access to communal areas differed among the hospitals. All 8 comprehensive stroke units had multidisciplinary stroke teams.

### Data collection

**Fig. 1:**
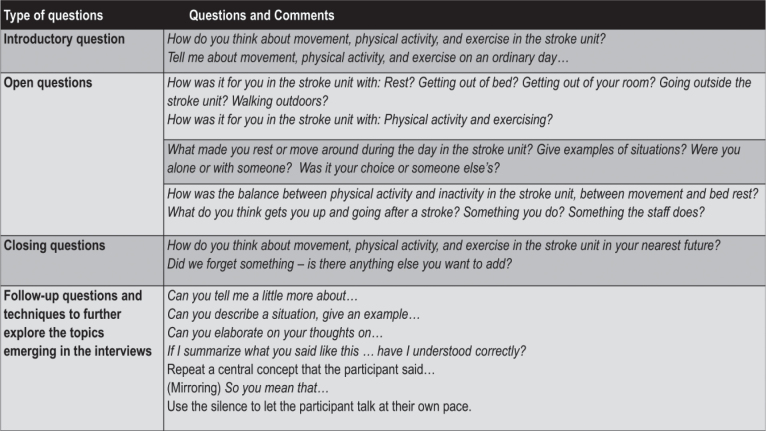
Interview guide.

A semi-structured interview guide (Fig. 1) was discussed and revised in collaboration with GH (30). MR conducted a pilot interview, and the in-depth individual interviews were conducted shortly after discharge (median 10.5 days) to avoid recall bias. To ensure that participants could speak freely, the interviews were conducted by a person not involved in the care and outside the hospital after discharge. Participants chose the time and place for the interviews, which was anywhere that was suitable for the purpose. Most participants were interviewed in their own home. Participants chose to have relatives nearby at 8 interviews, 3 of whom had relatives present in the same room; relatives were asked to not interact during the interview if they were not needed. MR conducted all interviews, which lasted from 25 to 60 min. Interviews were audio recorded and transcribed verbatim by MR and 2 research assistants. To facilitate communication and mutual understanding of concepts at the interviews, the moderator used synonyms for physical activity, inactivity, and exercise when appropriate. Field notes were not taken during the interviews. The 16 interviews provided rich and meaningful descriptions, with the dialogue revealing in-depth perspectives that adequately corresponded to the research question (19, 31). There was no need for further interviews.

### Data analysis

**Fig. 2:**
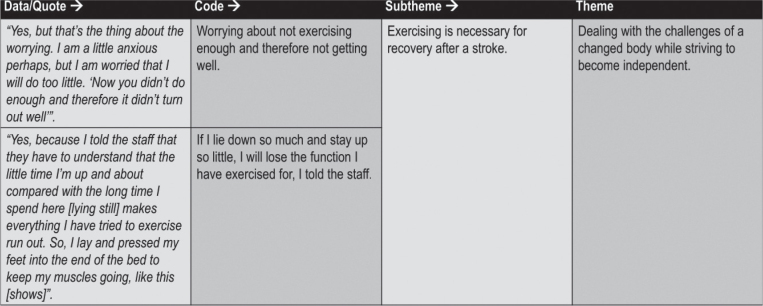
Examples of the coding process.

The transcribed interviews were subjected to a reflexive thematic analysis in 6 phases (18). In phase 1, 2 authors (MR and AP) individually became familiar with the data through listening, reading, and re-reading the interviews to gain a sense of the whole. In phases 2 and 3, initial codes were noted separately between the 2 authors, followed by joint coding and collaborative work in search of initial themes. In phase 4, the themes were reviewed, reorganized, and refined in collaboration with all 4 co-authors to reach consensus. In phase 5 the reflexive analysis moved back and forth between data, codes, and themes, as well as between parts of and the whole text and audio recordings to validate the linking between the themes and the interviews. GH validated the patient perspective in the analysis process. Participant quotes were selected in discussion among all 4 authors to best reflect the results of the analysis. In phase 6 MR wrote and reported the results. No software was used. The coding process is exemplified in Fig. 2.

## RESULTS

The thematic analysis resulted in 3 themes and their respective subthemes, as follows.

### Theme 1: “Dealing with the challenges of a changed body while striving to become independent”

**Fig. 3:**
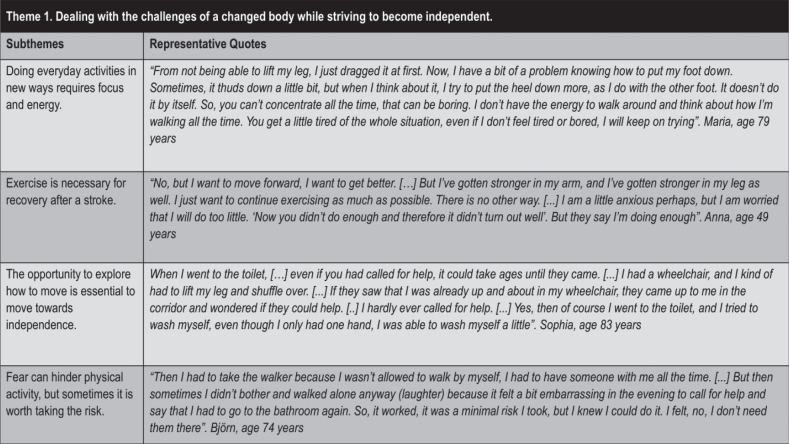
Theme 1 with subthemes and quotes.

In Theme 1 (Fig. 3), the participants described how the changed situation of falling ill and needing healthcare aroused a strong drive to continue to be an independent person who can manage on their own. Participants described how they did things in new ways and fought their fears to be able to move independently and get back to their old life and old self.

Within this theme, the subtheme “*Doing everyday activities in new ways requires focus and energy”* described how the participants experienced a changed body and ability to move. They had to manage their neurological symptoms and find strategies and solutions for doing habitual things in new ways and requiring concentration that could lead to fatigue.

Also within this theme, the subtheme “*Exercise is necessary for recovery after a stroke”* was derived, as many participants expressed that exercise was necessary to get better and make progress. They described taking the initiative to exercise on their own in different ways. Many said that exercise was required to get better, with a tone that expressed that exercise was a responsibility and signalled that those who do not exercise probably would never get better.

In the subtheme “*The opportunity to explore how to move is essential to move towards independence”*, the participants expressed that the ability to self-manage is fundamental, describing the opportunity and encouragement as positive to try to perform different physical and everyday activities. Needing help could bring experiences of gratitude and humbleness or shame and frustration. Situations arose in which staff and patients disagreed about the patient’s walking ability, resulting in agreement or conflict. Participants described finding it difficult to maintain integrity, and managing toilet visits was depicted as particularly important. Another reason to become independent was to avoid waiting a long time for help.

Finally, within this theme, the subtheme that “*Fear can hinder physical activity, but sometimes it is worth taking the risk”* was derived, as participants considered fear of falling to be the most common fear when moving. With a strong drive towards independence, participants sometimes tried to move on their own, even when staff did not recommend it because of the fall risk. Another fear of moving was related to anxiety about worsened symptoms, such as dizziness and nausea, or having a new stroke, such as in the event of severely increased blood pressure. Fear of COVID-19 was also a reason cited by patients for staying in their room.

### Theme 2: “The stroke unit is crucial for physical activity”

**Fig. 4:**
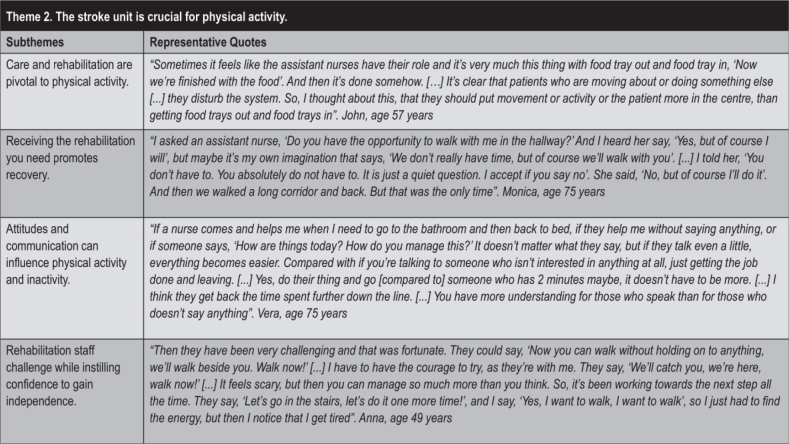
Theme 2 with subthemes and quotes.

In Theme 2 (Fig. 4), the participants emphasized that the stroke unit should provide the safety and courage that they needed to move after a stroke. This sense of security and empowerment determined if the participants undertook physical activity and if so, how much. They expressed a wish that the staff would support the patient to enable recovery and physical activity. The rehabilitation staff were perceived as having a more challenging and affirming role when the patients were physically active.

Within this theme, the subtheme “*Care and rehabilitation are pivotal to physical activity”* was derived as important, with participants expressing a need for care and rehabilitation to be individualized, with a holistic approach that took into account their background, situation, wishes, and resources. When the staff prioritized the needs of the patients over tasks and hospital routines, physical activity was promoted. Furthermore, when the staff was attentive to the patients’ needs and daily condition, it was appreciated. Participants moreover said that physical activities often involved close connections between patients and staff.

Also emerging within this theme was the subtheme that “*Receiving the rehabilitation you need promotes recovery”*. Everyday life in the stroke unit was described as a long line of “meal, bed rest, exercise, meal…”, often with many opportunities for exercise. However, several participants felt that they were not getting the rehabilitation they needed. Some described inactive days with “full service”, which was perceived as either positive or leaving them too passive. In addition, an improvement could result in more need for help, such as when a wheelchair user started to walk with help.

The subtheme “*Attitudes and communication can influence physical activity and inactivity”* also fell within this theme. Participants described how communication among rehabilitation staff, healthcare professionals, and patients influenced physical activity. Getting good information brought feelings of safety and involvement. Participants wanted to know the purpose of their exercises and to be given feedback about their recovery. When communication was lacking between the medical and rehabilitation staffs, participants had experiences of being told off by medical staff for walking independently despite having been cleared by rehabilitation staff to do so. Some respondents also expressed the perspective that the staff should be attentive and engaged in promoting rehabilitation and physical activity.

In expressing the subtheme that “*Rehabilitation staff challenge while instilling confidence to gain independence”*, participants said that they appreciated being pushed and challenged by the rehabilitation staff. Rehabilitation staff were a little tougher than medical staff, and sometimes pushed patients to exercise at the limit of their ability, according to participants. This encouragement was perceived as the rehabilitation staff speeding up the process towards a higher degree of independence.

### Theme 3: “Physical activity is important for interaction with others, autonomy, and feeling seen”

**Fig. 5:**
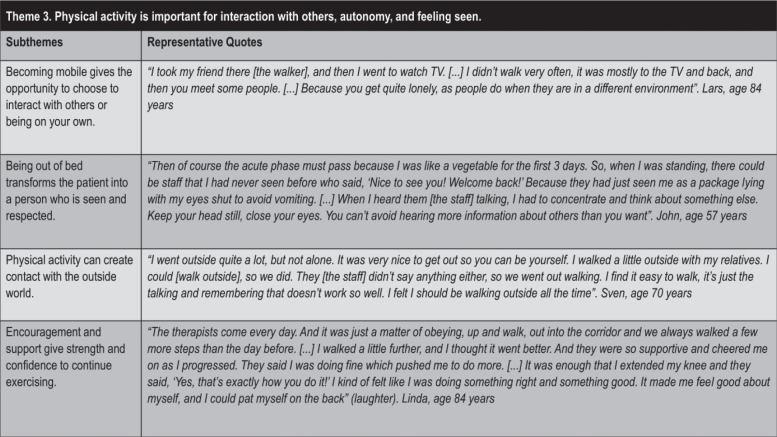
Theme 3 with subthemes and quotes.

In theme 3 (Fig. 5), the participants described how physical activities influenced how they thought about themselves and related to others. They described the feeling that by being physically active they would be seen and encouraged. This experience created confidence in their own abilities and contributions to their autonomy. In contrast, staying in bed was described as resulting in feelings of passivity and invisibility, as the nursing staff were busy keeping up with their everyday jobs.

Within this theme, the subtheme that “*Becoming mobile gives the opportunity to choose to interact with others or being on your own*” was derived through observations that mobility allowed for a choice between community or privacy, whereas immobility could cause loneliness and isolation. Being left alone in the room for some peace and quiet was perceived as nice, as many participants described symptoms of fatigue that necessitated rest, especially early after acute stroke. Increased mobility and independence drew participants more to communal areas, such as television rooms and dining rooms outside the patient room.

According to the participants, “*Being out of bed transforms the patient into a person who is seen and respected*”. They described being seen and respected when up and about. Being up and moving created natural opportunities to greet and meet others and affected how the participants perceived their treatment by the staff. Getting out of bed meant being seen as a person. Participants expressed that they stayed in bed to avoid being a bother to the staff, who seemed to have a lot to do and needed to prioritize other patients. Strategies that participants described for dealing with these impressions included becoming one with a grey wall or closing one’s eyes to disconnect from the situation.

Participants indicated that “*Physical activity can create contact with the outside world*” and described how experiences with physical activity mediated contact with the outside world, such as with nature when walking past a window or sitting on the balcony of the ward. Participants described the importance of physical activity for connecting with the outside, following the rhythms of nature, and broadening their horizons. Being able to go out gave a feeling of being more alive and of expanded views and normalcy.

Finally, within this theme, participants expressed that “*Encouragement and support give strength and confidence to continue exercising*”, as having a stroke could be experienced as tough and transformative. Participants described how support from healthcare professionals, rehabilitation staff, other patients, and relatives was important for instilling hope and energy. Struggling in everyday life and with exercise could weaken self-confidence in their abilities, and some participants expressed that confirmation from others about their progress supported their self-motivation and self-confidence.

## DISCUSSION

The analyses of the interviews produced 3 overarching themes describing physical activity and inactivity in the stroke unit from the patients’ perspective.

In Theme 1 “*Dealing with the challenges of a changed body while striving to become independent*”, participants described how to cope with their symptoms, functional ability, and a new situation when finding new ways to move and function. In addition, participants expressed the conviction that exercise is necessary to get better.

Theme 2 “*The stroke unit is crucial for physical activity*” included experiences of rehabilitation with needs being met or not, a desire to be involved in one’s care and rehabilitation, and the need for staff to centre patients.

Theme 3 “*Physical activity is important for interaction with others, autonomy, and feeling seen*” encompassed the participants’ characterizations throughout the interviews of the value of movement as going beyond physical activity. Being up on one’s feet was perceived as vital for feeling seen and being respected as a person. Being able to move was also felt to influence self-image, relating to others, and how others relate back.

The participants in this study emphasized a strong drive towards independence after stroke. They called for an active everyday life in the stroke unit, where the exercise that patients need is provided. This rehabilitative approach has been described in a Finnish study where patients received support for their physical functioning, independence, and self-confidence (32). In that study, more physical activity in the everyday routine and exercise was achieved with a rehabilitative approach including an explicit awareness, way of work, mutual conceptions, and a tight multi-professional team (32). In addition, in the current study, a narrative was derived of the participants coping with their changed body and taking charge over their exercise and situation as capable persons. Participants also expressed the desire for a more holistic view from care providers, and to be seen as a person and not a diagnosis. This corresponds to the holistic bio-psycho-social model where the biological, psychological and social contexts are equally included (33). In addition, participants described the need to be listened to and to receive individualized care and rehabilitation. Listening to the patient’s narrative and regarding the patient as a person capable of being involved in decision-making about and planning of their own care and rehabilitation aligns with the person-centred care approach (4, 34). Some of the statements from participants in this study are in line with the concept of person-centred care, which is supported by the Swedish national stroke guidelines. The participants also described how they perceived movement as vital for feeling seen and being respected as a person. Similar results were reported in a qualitative study of patient experiences after hip surgery, in which respondents described the need to be seen as a person and to have other needs met, and the drive for independence when cared for in a geriatric ward (35). It can be assumed that some of the findings of the current study can be applied for diagnoses other than stroke and specialized wards other than stroke units. Nevertheless, this qualitative study is focused on providing a contextualized understanding of the patient experience without an effort to generalize. The participants’ feelings of being seen when moving their body can be explained by the theory “phenomenology of the body” (36). According to this theory the world is experienced through the moving body, the lived body. In the current study the participants expressed that being up on their feet and moving meant having contact with the world, and others expressed that staying in bed resulted in the opposite.

### Study limitations

Limitations of this study include potential influence from the authors, with a researcher bias arising from preconceptions. Two of the 4 authors are physiotherapists with a preference for physical activity. Another limitation is the risk of recall bias because the participants were interviewed 1–2 weeks after discharge and may have had difficulties remembering their experiences. The risk of selection bias was addressed with a careful, purposeful selection of participants to assure a heterogeneous and representative study sample. To support dependability of the results, a carefully constructed interview guide was used at the interviews, which all were conducted by the same moderator. To further strengthen the dependability of the results, all interviews were conducted over a short time-period to reduce the risk of contextual changes that could have interfered with participant experiences (19). However, interpretation of the results should be made with consideration of the Swedish stroke care context and of the pandemic situation in Sweden in the autumn of 2022.The authors’ preconceptions are reported in the study, a transparency that can increase the confirmability of the results (19). The detailed descriptions of methods and analysis procedures are intended to increase the transferability of the findings (19). To strengthen the credibility of the study, the participants’ quotes were carefully selected by all co-authors to reflect the results and support the analysis.

Increasing physical activity is a priority for people after stroke in order to increase function and reduce the risk of stroke recurrence (37). Previous studies have focused on objective and subjective assessments for physical activity, although patients consider measuring goal achievement and the impact of physical activity on fatigue to also be important (37). Patients’ perspectives on stroke rehabilitation should be considered in quality-improvement efforts. Further research can broaden perspectives on physical activity in the stroke unit, and interviews with healthcare and rehabilitation staff could provide new insights. In addition, many persons are less physically activity after stroke, and qualitative research could bring new understanding about the factors involved. Even though movement is essential at a stroke unit as part of the rehabilitation and everyday activities for patients with acute stroke, there is often a dissonance between the ambitions of the staff, the visions in guidelines, and clinical reality. Good intentions get lost with staffing shortages and lack of continuity. International and Swedish policy implications and guidelines on stroke care are, in many ways, congruent with some of the statements being made by the interviewed participants in this study. However, attitudes, dialogue, partnership, and other soft values are not included in guidelines and programmes, even though they exist every day in stroke unit care and rehabilitation (38). The current findings will be helpful for improving the experiences for patients at stroke units, and for improving the quality of stroke unit care. To increase knowledge and raise awareness about different aspects of stroke unit care is necessary in a multi-disciplinary stroke team where the learning process is never completed. Further collaboration with stakeholders and the inclusion of patients’ and relatives’ perspectives are needed in both research and clinical practice.

### Conclusion

Patients regard movement as more important than physical activity in the stroke unit, as it involves being seen and respected. Patients recovering from stroke try to deal with their changed body and abilities while striving to regain independence, and they express that the way to independence is through exercise. In addition, patients want to be involved in their own care and to be supported by the staff to promote physical activity. Participants want care and rehabilitation to be individualized in the stroke unit to meet their need for rehabilitation, help, and rest. Physical activity is considered as offering the possibility of choosing between community and being alone, and it influences connecting with others and the outside world.
